# A scoping review protocol on digital health equity among immigrant populations: Planning and managing effective digital health services

**DOI:** 10.1371/journal.pone.0357612

**Published:** 2026-09-10

**Authors:** Salsabela Razaq, Saleema Allana

**Affiliations:** Arthur Labatt Family School of Nursing, Western University, London, Ontario, Canada; University of Coimbra: Universidade de Coimbra, PORTUGAL

## Abstract

Equity is central to the effective planning and management of digital health services, particularly as technology adoption rises across Canada and the United States. Immigrant populations, despite representing a significant portion of the population and being considered an underserved group, continue to experience digital health inequities. To support the planning and management of effective digital health services, this protocol outlines a scoping review designed to map the existing research on digital health equity among immigrant populations. A comprehensive understanding of the current literature is imperative to inform digital health management and policies. The five-stage methodological framework outlined by Arksey and O’Malley will be used to guide this scoping review protocol. It is anticipated that the finding will highlight specific barriers to digital health equity among immigrant populations, providing the necessary guidance for future digital health planning and management. The scoping review protocol is registered with the Open Science Framework, registration: osf.io/ugv4d.

## Introduction

The COVID-19 pandemic accelerated the adoption of digital health technologies within healthcare systems across the globe [1,2]. Digital health technologies are tools, services, or platforms used to deliver healthcare or facilitate health management [3]. Electronic medical records, mobile health applications, and patient portals are some examples of digital health technologies [3]. Although digital health technologies can be leveraged to improve patient health outcomes, they can simultaneously create or worsen digital health inequities [4,5]. Populations that are immigrants are especially vulnerable to digital health inequities [6–8].

People who are immigrants are recognized as an underserved group [6–9], that experience significant barriers participating in society [9]. Addressing the barriers experienced by those who are immigrants is critical given the rapid growth of this population over the last decade [10–12]. The United States (US) ranks first globally with 50.6 million immigrants residing in the country [13]. Canada also ranks among the top ten countries, with over 8 million immigrants that account for approximately 2

5% of the total population [13,14]. Consequently, digital health planning, management, and polices must prioritize this demographic to prevent and mitigate inequities.

Unfortunately, there is a notable gap in the literature exploring digital health equity among populations that are immigrants. Although previous reviews have explored digital health equity in broad terms [15], no review specifically focuses on populations that are immigrants. A scoping review that maps the existing literature on digital health equity among people who are immigrants can address this gap, and guide future research directions.

### Digital health equity

Digital health equity is described as the fair opportunity to benefit from digital health technologies [16]. Within digital health equity research, ‘digital equity’ and ‘health equity’ commonly form the foundational understanding of digital health equity [15]. Digital equity as “the condition in which individuals and communities have the information technology capacity that is needed for full participation in the society and economy” (15 p. 933). Health equity as “the attainment of the highest level of health for all people that requires valuing everyone equally with focused and ongoing societal efforts to address avoidable inequalities, historical and contemporary injustices, and the elimination of health and health care disparities” (15 p. 933).

Promoting digital health equity is multifaceted; the Conceptual Framework of Digital Inclusion outlines three core components that can directly influence digital health equity [17]. These three components include digital access, digital literacy, and digital acceptance [17]. The Conceptual Framework of Digital Inclusion will guide the understanding of digital health equity within this scoping review protocol (see the theoretical framework section of this paper).

### Digital health inequities among immigrants

The digital divide is a primary driver of digital health inequities, stemming from a lack of digital access and literacy [4,18–20]. The Conceptual Framework of Digital Inclusion suggests that accessibility and literacy significantly influence digital health equity [17]. Those who are immigrants are reported to experience digital health inequities related to access and literacy challenges [2,21]. For example, people who are immigrants residing in Canada are more likely to experience internet accessibility challenges when compared to their non-immigrant counterparts [22], due to financial constraints that restrict access to high-speed internet [5]. Similarly in the US, 12% of people from immigrant backgrounds do not have internet access in their home [23]. Moreover, those with limited English proficiency, have poor digital literacy skills, which directly impacts navigating digital health technologies [24–26]. For example, approximately 80% of Canadian individuals who are immigrants require digital literacy training [27] and 33% of US workers who are immigrants report a lack digital skills [28].

### Study purpose and objectives

This scoping review will map the existing research on digital health equity among populations that are immigrants in Canada and the US in relation to digital access, literacy and acceptance. The specific objectives are to: (1) identify the gaps in the current digital healthcare landscape; and (2) recommend future directions for planning and managing effective digital health services.

### Theoretical framework

The Conceptual Framework of Digital Inclusion identifies digital accessibility, digital literacy, and digital acceptance as the necessary components for achieving digital inclusion. Digital accessibility is characterized by the availability of digital tools, internet connectivity, affordability, and assistive design [17]. Digital literacy encompasses computer literacy (skills to operate digital tools) and information and media literacy (skills to find, evaluate, and use information from digital tools) [17]. Digital acceptance includes awareness of technology, perceived usefulness, perceived usability, and perceived trustworthiness, which impact an individual’s attitudes towards utilizing digital technology [17].

The Conceptual Framework of Digital Inclusion is fundamental to any digital engagement, including digital health technologies [17]. The Conceptual Framework of Digital Inclusion will underpin this scoping review because the framework is directly relevant and applicable to digital health equity. The framework was developed through the understanding that digital tools can be of immense value, but these same tools can cause or exacerbate inequities [17] directly aligning with digital health equity research [25,29,30].

The Conceptual Framework of Digital Inclusion allows for a structured understanding of digital health equity within populations who are immigrants. This scoping review will define digital health equity by the presence of the three core components (digital accessibility, digital literacy, and digital acceptance). The framework’s three components will be used within the search strategy to locate relevant studies. Data collection will involve extracting digital accessibility, digital literacy, and digital acceptance data. Inequities experienced by populations that are immigrants will be classified within these categories. Through this structured categorization, this scoping review will identify implications that can promote digital health equity.

## Materials and methods

### Study design

A scoping review will be conducted to uncover what is known within the existing research regarding digital health equity among populations who are immigrants in Canada and the US. It is anticipated this scoping review will be completed by June 2026.This review will follow the five-stage methodological framework outlined by Arksey and O’Malley [31]. The first stage involves identifying the research question. The second stage identifies relevant studies. The third stage involves study selection. The fourth stage entails charting of the data. The fifth and final stage involves collating, summarizing, and reporting results [31]. Arksey and O’Malley's framework was selected because it facilitates a clear and structured way to effectively map complex fields of study, such as digital health equity [31,32]. This framework was also selected for its iterative process, which permits the refinement of the search strategy as familiarity with the literature increases. This flexibility is essential for mapping the literature regarding digital health equity [33]. This scoping review protocol will further adhere to the Preferred Reporting Items for Systematic review and Meta-Analysis Protocols (PRISMA-P) checklist, see S1 Checklist [34].

### Stage 1: Identifying the research question

The research question that will be explored in this scoping review is as follows: “What is known within the existing research regarding digital health equity (in relation to digital access, literacy, and acceptance) among communities that are immigrants in Canada and the US?” The research question was developed based on the Population, Concept, and Context (PCC) Framework. The population are immigrants, the concept is digital health equity, and the context is within Canada and the US. This research question is of vital importance because it has been proven that although digital health technologies can benefit patient health outcomes, they also risk worsening patient health outcomes [4,5].

Populations that are immigrants will be the focus of this review because they are more vulnerable to digital health inequities relative to non-immigrants and they face unique barriers to digital health that distinguish them from other underserved groups [10].While digital health inequities exist for several equity-denied groups, those that are immigrants experience distinct barriers. Digital health technologies are rarely optimized for the diverse linguistic needs of this group, frequently lacking interfaces in the patient’s preferred language [5]. Additionally, a lack of culturally tailored training or technical support prevents those who are immigrants to confidently navigate complex platforms. For many individuals that are immigrants, a profound mistrust in digital health technologies is caused by fears related to data privacy and legal repercussions [5]. These inequities have become increasingly pronounced following the COVID-19 pandemic, which accelerated the adoption of digital health technologies [1,2,6–8]. Consequently, this acceleration exacerbated inequities experienced by the growing populations who are immigrants in Canada and the US, risks their exclusion to essential healthcare services [10]. Moreover, Canada and the US will be the geographical focus for several reasons. Both countries have rapidly adopted digital health technologies within their healthcare systems as described in the introduction of this protocol. Both countries share similar digital infrastructure and face comparable challenges related to the digital divide [35]. Canada and the US are also both prominent immigrant-receiving countries [13]; therefore, focusing on these two countries is appropriate based on their shared contexts.

### Stage 2: Searching the literature

The electronic databases PubMed, CINAHL, Medline Ovid, and Scopus will be used in the scoping review. To avoid publication bias, and for the purposes of obtaining grey literature, Pro Quest Dissertations will also be included. Expert guidance will be sought through consultation with a qualified Western University librarian to optimize the search strategy. The term ‘digital health equity’ will be broken down into the three core components of the Conceptual Framework of Digital Inclusion (access, literacy, and acceptance). The term ‘immigrant’ will include synonyms identified within relevant literature. The final search strategy will utilize a combination of keywords, Boolean operators, truncation, subject headings, and MESH terms, to maximize results (See S2 Appendix).

The search will not include the geographical location to prevent the possibly of inadvertently excluding relevant studies. Rather, the geographical location will be manually filtered during the abstract and full-text screening of studies. Since digital health equity is an emerging and evolving research area [1,2], no date filters were applied in order to capture the field’s full trajectory.

A search validation procedure will involve an initial search string that encompasses keywords based on the PCC Framework. This search string will be conducted in each database and the first 25 citations from each database will be reviewed for relevance. Relevance will be determined by comparing the title, abstract, and keywords to the PCC framework. Of the studies deemed relevant, additional keywords will be identified and included within the final search strategy. This is not a living review; the literature will be searched at one point in time to capture the available evidence up until the date of the final search.

### Stage 3: Screening the articles

The studies will be screened based on the inclusion and exclusion criteria. The inclusion criteria will be as follows: (1) a digital health technology must be included; (2) individuals who are immigrants must be the population of interest, either stated explicitly or evident based on country of birth; (3) the content must explore at least one aspect of digital health equity according to the Conceptual Framework of Digital Inclusion (access, literacy, acceptability); and (4) the research must be a qualitative, quantitative, or mixed methodology primary study (it can be published or unpublished). Studies will be excluded if they (1) are not in English; (2) include populations that are non-immigrants without separating findings for immigrant groups; and (3) if the full-text study is not retrievable. The review will only incorporate English studies because the protocol is part of a master’s thesis that will be conducted without funding; therefore, translation services are not a feasible option. Justification for the inclusion and exclusion criteria is based on the purpose of the review, the PCC framework, and the Conceptual Framework of Digital Inclusion.

Two independent reviewers will screen studies using the Covidence platform in two stages [31]. In the first stage, title and abstract screening, reviewers will exclude studies meeting any exclusion criteria, while retaining those that meet the inclusion criteria. In the second stage, full-text screening, the study is to be read in full. Studies will only be included if they satisfy all inclusion criteria and meet no exclusion criteria [31]. Conflicts will be resolved through a consensus-based conversation between the two reviewers. A third reviewer will be available to mediate any unresolved conflicts. Inter-rater reliability will be reported as a percentage. Given the scoping nature of this review, screening will not be blinded.

### Stage 4: Extracting the data

A data-charting form will be created following a similar format proposed by Arksey and O’Malley [31]. To provide a comprehensive contextual understanding, the form will capture key study characteristics. The data-charting form will include the author(s), year of publication, study location, type of digital health technology, study population, aims of the study, and methodology. Additionally, findings related to digital health equity will be extracted according to the Conceptual Framework of Digital Inclusion (digital access, literacy and acceptance). Any unanticipated data will be recorded as other important findings. The data-charting form will be iteratively adjusted if new variables emerge [31].

Data extraction will be completed by the primary investigator. To ensure reliability, the supervisor will verify the extraction of a random sample of included studies. Inter-rater reliability will be documented as percentage of agreement. Any conflicts will be resolved through a discussion between the primary investigator and the supervisor, with a third reviewer consulted if a consensus is not reached.

### Stage 5: Analyzing and reporting the data

The extracted data will undergo both a numerical and thematic analysis. First, a numerical analysis will summarize the included studies’ characteristics using Excel-generated tables. These tables will report the frequencies of study locations, immigrant groups, types of digital health technologies utilized, and research methodologies [31]. This analysis plan is linked with the PCC Framework as we plan to analyze the data around which immigrant groups were studied in the included articles and where those studies were conducted. Second, a thematic analysis will explore the qualitative findings using a deductive and inductive approach. Initially, a deductive approach guided by the Conceptual Framework of Digital Inclusion will categorize findings into the framework’s three core components (digital access, literacy, and acceptance). An inductive approach will allow for the identification of emerging themes not captured by the Conceptual Framework of Digital Inclusion. The emerging categories will then be categorized under the broader themes [31]. The analysis plan considers the Conceptual Framework of Digital Inclusion by highlighting the findings related to digital access, literacy, and acceptance [17].

Missing data is not anticipated, as study inclusion requires at least one component of the Conceptual Framework of Digital Inclusion to be addressed. Collectively, the included studies will cover all aspects of the framework. To ensure data validation, the data-charting form is designed to capture all relevant elements of the research question with particular attention to the PCC Framework.

To ensure clarity and comprehensiveness, the primary investigator will pilot-test the data-charting form on a sample of the included studies, subject to review by the supervisor. Any necessary adjustments identified during this pilot will be incorporated into the final form. The Mixed Methods Appraisal Tool will be used to assess the quality of the included studies. The quality assessments will be used to contextualize the findings but, no studies will be excluded based on these results.

Blinding will not be utilized at this stage, as the primary investigator is a student who must be involved in every stage of the scoping review. To ensure reliability, the supervisor will cross-check a random sample of the primary investigators results. Inter-rater reliability will be calculated and reported as a percentage of agreement. Any discrepancies will be resolved through a consensus-based discussion, with a third reviewer consulted if conflicts remain unresolved.

## Discussion

As previously noted, immigration is on the rise [11], creating a growing population that is disproportionately vulnerable to digital health inequities [7,10]. Simultaneously, digital health technologies are becoming a central modality within health systems in Canada and the US [1]. Despite the potential of digital health technologies to improve care, those who are immigrants often experience significant health decline within their first two years of settlement [36]. Recent studies highlight that digital health inequities among communities that are immigrants remain significantly understudied and warrant greater scholarly attention [2,37,38]. This scoping review will address this gap by identifying relevant literature on digital health equity among populations that are immigrants in Canada and the US.

The review will provide a comprehensive overview of the current state of digital healthcare, while simultaneously identifying critical gaps where the needs of people who are immigrants remain overlooked. These findings can directly inform the future direction of digital health planning, management, and policy development. Specifically, insight into distinct inequities can guide the implementation of inclusive interventions to proactively foster digital health equity. Finally, this review can shape digital health polices, thereby sustaining digital health equity for populations that are immigrants.

## Supporting information

S1 Checklist PRISMA-P 2015 checklist. (DOCX)

S2 Appendix Search strategy. (DOCX)
