# Supplementary material for: A scoping review protocol on digital health equity among immigrant populations: Planning and managing effective digital health services
Source: PLoS One. 2026 Sep 10;21(9):e0357612. doi: 10.1371/journal.pone.0357612 (PMC13561313; doi:10.1371/journal.pone.0357612)
Supplement: Supplementary file 2 [file pone.0357612.s002.docx]

# Appendix A

## Search Strategy

A draft search strategy for Medline Ovid is as follows:

( ("digital health*" OR "virtual care" OR "virtual health" OR "eHealth*" OR

"mHealth*" OR "telemedicine" OR "telehealth" OR "mobile health" OR

"mobile app*" OR "wearable*" OR "remote monitor*" OR "electronic health record*" OR

"patient portal*")).tw,kf.

AND

(("equit*" OR "disparit*" OR "inequalit*" OR "barrier*" OR "access*" OR

"adopt*" OR "accept*" OR "trust" OR "bias*" OR "justice" OR

"safety net*" OR "community health*" OR "insure*" OR "uninsured" OR

"afford*" OR "cost*" OR "resource*" OR "socioeconomic" OR "SES" OR

"poverty" OR "social determinants" OR "SDOH" OR "racis*" OR

"discriminat*" OR "vulnerab*" OR "perceived useful*" OR

"perceived usabili*" OR "aware*" OR "connect*" OR "avail*" OR

"digital determinants of health" OR "digital divide*" OR "digital inclusion" OR

"digital access" OR "digital literacy" OR "computer literacy" OR

"assistive design")).tw,kf.

AND

((immigrant* OR newcomer* OR refugee* OR "temporary resident*" OR migrant* OR

OR "first generation immigrant*" OR racialized OR "equity deserving*").tw,kf.

OR Exp “Emigrants and Immigrants”/ OR Exp Refugees/ OR

exp “Minority Groups”/ OR exp “Vulnerable Populations”/ OR exp “Undocumented Immigrants”/
